# Condomless receptive anal intercourse is associated with markers of mucosal inflammation in a cohort of men who have sex with men in Atlanta, Georgia

**DOI:** 10.1002/jia2.25859

**Published:** 2021-12-15

**Authors:** Colleen F. Kelley, Ilana Pollack, Rami Yacoub, Zhengyi Zhu, Vanessa E. Van Doren, Sanjeev Gumber, Rama R. Amara, Veronika Fedirko, Colleen S. Kraft, Tom J. B. de Man, Yi‐Juan Hu, Cassie Grimsley Ackerley, Patrick S. Sullivan, Roberd M. Bostick

**Affiliations:** ^1^ Division of Infectious Diseases Department of Medicine Emory University School of Medicine, The Hope Clinic Atlanta Georgia USA; ^2^ Department of Epidemiology Rollins School of Public Health Emory University Atlanta Georgia USA; ^3^ Department of Biostatistics and Bioinformatics Rollins School of Public Health Emory University Atlanta Georgia USA; ^4^ Division of Pathology Yerkes National Primate Research Center Emory University Atlanta Georgia USA; ^5^ Department of Pathology and Laboratory Medicine Emory University School of Medicine Atlanta Georgia USA; ^6^ Division of Microbiology and Immunology Yerkes National Primate Research Center Emory University Atlanta Georgia USA; ^7^ Department of Microbiology and Immunology Emory University School of Medicine Atlanta Georgia USA; ^8^ Winship Cancer Institute Emory University School of Medicine Atlanta Georgia USA; ^9^ MilliporeSigma Rockville Maryland USA

**Keywords:** men who have sex with men, rectal mucosa, HIV transmission, receptive anal intercourse, mucosal immunology, microbiome

## Abstract

**Introduction:**

We previously showed that the rectal mucosal immune environment among men who have sex with men (MSM) engaging in condomless receptive anal intercourse (CRAI) is immunologically distinct from that of men who do not engage in anal intercourse (AI). Here, we further examined these differences with quantitative immunohistochemistry to better understand the geographic distribution of immune markers of interest.

**Methods:**

We enrolled a cohort of MSM engaging in CRAI (*n* = 41) and men who do not engage in AI (*n* = 21) between October 2013 and April 2015. Participants were healthy, HIV‐negative men aged 18–45 from the metro Atlanta area. We performed rectal mucosal sampling via rigid sigmoidoscopy during two study visits separated by a median of nine weeks and timed with sexual activity for MSM engaging in CRAI. We used standardized, automated immunohistochemistry and quantitative image analysis to investigate the rectal mucosal distribution of neutrophils (MPO), IL‐17‐producing cells (IL‐17) and T_regs_ (FOXP3) in the lamina propria, and cellular proliferation (Ki67) and adherens junction protein (E‐cadherin) in the epithelium. We examined associations between biomarker expression and the rectal mucosal microbiota composition by 16s rRNA sequencing.

**Results:**

Relative to the colonic crypt base, IL‐17, FOXP3, and MPO expression increased towards the rectal lumen, while Ki67 decreased and E‐cadherin was more uniformly distributed. Throughout the rectal mucosa distribution examined, MSM engaging in CRAI had higher mean lamina propria MPO expression (*p* = 0.04) and epithelial Ki67 (*p* = 0.04) compared to controls. There were no significant differences in IL‐17, FOXP3 or E‐cadherin expression. We found no significant associations of the five biomarkers with the global rectal microbiota composition or the individual taxa examined.

**Conclusions:**

Understanding the mucosal distribution of inflammatory mediators can enhance our knowledge of the earliest events in HIV transmission. Neutrophil enrichment and crypt epithelial cell proliferation likely represent sub‐clinical inflammation in response to CRAI in the rectal mucosa of MSM, which could increase the risk for HIV acquisition. However, the contributory role of the microbiota in mucosal inflammation among MSM remains unclear. HIV prevention may be enhanced by interventions that reduce inflammation or capitalize on the presence of specific inflammatory mechanisms during HIV exposure.

## 1 INTRODUCTION

From 2013 to 2017 in the United States, 72% of new HIV infections occurred among men who have sex with men (MSM), with approximately 70% attributed to rectal mucosal exposure during receptive anal intercourse (AI) [1, 2]. HIV transmission probability per exposure event is 18‐fold and 50‐fold higher for rectal compared to vaginal or penile exposure, respectively [3]. There are many potential contributors to this higher transmission risk. The rectal mucosa is composed of single‐layer columnar epithelium, which may be more susceptible to mechanical microtrauma during intercourse than the stratified squamous epithelium that lines most of the penis and vagina [4]. The gut, particularly the distal rectum where HIV transmission is most likely to occur during AI, also contains most of the body's lymphocytes, many of which are primary HIV target cells [5, 6].

Understanding rectal mucosal HIV transmission biology is essential for designing biomedical prevention interventions, including an effective vaccine; however, the rectal mucosa has been understudied to date. Penile and vaginal neutrophil activity has been associated with susceptibility to HIV infection [7, 8, 9]. This is thought to be partially due to inflammation‐related epithelial barrier defects [10]. IL‐17‐producing cells, including γδ T cells, MAIT, NK cells and NK‐T cells, are well‐represented at injury and inflammation sites and expand at mucosal surfaces to help clear invading pathogens and protect barrier integrity [11]. IL‐17‐producing CD4+ T cells (Th17) are primary HIV targets, and mucosal anti‐inflammatory FOXP3+ T regulatory cells (T_reg_) suppress inflammation and tissue damage [12, 13]. The intercellular junctions comprising mucosal epithelial barriers also respond to inflammation, leading to increased permeability through inflammatory cytokine release [14]. Epithelial integrity and mucosal immune cell composition play critical roles in host defence and pathogen susceptibility and should be examined in the rectal mucosa, specifically, to better define their role in rectal HIV transmission.

Our 2017 study was among the first to evaluate human rectal mucosal immunology in MSM [15]. We found that the rectal mucosa of MSM engaging in condomless receptive anal intercourse (CRAI) when compared to men who did not engage in AI was enriched for Th17 cells and proliferating and pro‐inflammatory cytokine‐expressing CD8+ T cells and demonstrated higher expression of genes associated with mucosal injury and repair, especially after recent intercourse. This is consistent with prior evidence that neutrophils and IL‐17‐producing cells are important inflammatory mediators within the gastrointestinal (GI) mucosa [16, 17]. We also discovered a microbiome that was enriched for the *Prevotellaceae* over *Bacteroidaceae* family, and in another study [18], that repeated rectal hyperosmolar lubricant application was associated with a microbiome shift favouring *Prevotellaceae* over *Bacteroidaceae*. We hypothesize that this distinct rectal mucosal immune milieu could be attributed to repeated mucosal immune responses to intercourse with antigenic mucosal exposure to the gut microbiome during intercourse and/or semen exposure, and/or rectal product use [19, 20].

The flow cytometry methods employed in our prior study could not be used to examine either the epithelial border or tissue distributions of specific rectal mucosal cellular subsets. Thus, in the study presented herein, we used standardized, automated immunohistochemistry (IHC) and quantitative image analysis to investigate tissue distributions of two biomarkers in the rectal mucosal crypt epithelium, E‐cadherin (adherens junction protein) and Ki67 (proliferation marker), and three cellular markers in the lamina propria, MPO (neutrophils), IL‐17 (mucosal pro‐inflammatory cells) and FOXP3 (T_reg_), among MSM who engaged in CRAI compared to men who did not engage in AI. We examined temporal differences in biomarker expression between study visits to explore whether CRAI timing influenced biomarker expression, overall differences in biomarker expression between cases and controls and the spatial relationship of these biomarkers relative to the rectal lumen. Finally, we examined biomarker associations with gut microbiota composition to evaluate whether or how the gut microbiota may influence rectal HIV transmission in humans engaging in CRAI. Our results provide insight into the potential mechanisms of rectal mucosal HIV transmission.

## 2 METHODS

The previously described MSM and control cohorts both comprised healthy, HIV‐negative men aged 18–45 from the metro Atlanta area enrolled between October 2013 and April 2015. The MSM cohort engaged in CRAI with a monogamous, HIV‐negative partner at least four times monthly. The control cohort had never engaged in AI [15]. Demographic and clinical characteristics are presented in Table 1.

**Table 1:** Demographic and clinical characteristics of the study groups

Characteristic^a^	MSM engaging in CRAI (*n* = 41)	Men not engaging in AI (*n* = 21)	*p*‐Value^b^
Median age in years (25th, 75th)	28.0 (26.0, 34.0)	24.0 (24.0, 30.0)	0.02
Race *n* (%)			
White	33 (80.5)	14 (66.7)	–
Black	6 (14.6)	2 (9.5)	–
Other	2 (4.9)	5 (23.8)	0.11
Lubricant use *n* (%)	39 (95.1)	NA	–
Enema use *n* (%)	18 (43.9)	NA	–
Median CRAI episodes in previous month (25th, 75th)	5.00 (5.00, 8.00)	NA	–

Abbreviations: AI, anal intercourse; CRAI, condomless receptive anal intercourse; MSM, men who have sex with men.

^a^ These variables were measured at study visit 1.

^b^ *p*‐Values represent Wilcoxon rank‐sum test for continuous variables and the Fisher's exact test for categorical variables.

### 2.1 The clinical cohort

The study entailed three visits. At an initial screening visit, eligible participants completed informed consent, a brief medical and sexual history, physical examination, rapid HIV testing and blood collection. One to six weeks later, participants underwent peripheral blood collection and rectal biopsy sampling via rigid sigmoidoscopy. MSM participants were asked to abstain from CRAI >72 hours before this visit. All participants returned for a third visit and again underwent peripheral blood and rectal biopsy sampling 8–16 weeks after visit 1. MSM participants were asked to engage in CRAI <24 hours before this visit and keep a record detailing each CRAI episode [21], including condom use, if ejaculation occurred during intercourse and lubricant and/or enema use. The Institutional Review Board at Emory University approved this study (#IRB00067782).

### 2.2 Quantitative immunohistochemistry and quantitative image analysis

Three rectal biopsies were oriented on a strip of bibulous paper in normal saline, transferred to 10% normal‐buffered formalin for 24 hours and stored in 70% ethanol prior to paraffin block embedment. Five slides with three levels of 3 μm‐thick biopsy sections taken 40 μm apart were prepared for each biomarker. Antigens were retrieved using a Lab Vision PT Module in 1:100x pH 6.0 citrate buffer (ThermoScientific, TH 250‐PM1X). Slides were immunohistochemically processed in a DakoCytomation Autostainer Plus System (Agilent Dako, Santa Clara, CA) using a labelled streptavidin‐biotin kit (Thermo Fisher Scientific UltraVisionKit, TP‐125‐HL; Thermo Fisher Scientific, Fremont, CA). The five biomarker antibodies were anti‐IL‐17 polyclonal goat antibody (R&D Systems, Cat. #AF‐317‐NA, RRID: AB_354463) at a 1:100 dilution; anti‐FOXP3 mouse monoclonal antibody, clone (236A/E7) (Abcam, Cat. #ab20034, RRID: AB_445284) at a 1:300 dilution, anti‐myeloperoxidase rabbit polyclonal antibody (Agilent, Cat. #A039829‐2, RRID: AB_2335676) at a 1:1500 dilution; anti‐E‐cadherin mouse monoclonal antibody, clone (4A2C7) (ThermoFisher, Cat. # 33–4000, RRID: AB_2533118) at a 1:150 dilution; and anti‐Ki67 mouse monoclonal antibody, clone (mib‐1) (Agilent, Cat. #M724001‐2, RRID: AB_2631211) at a 1:350 dilution. Each batch included positive (human tonsil tissue for FOXP3, Ki67 and MPO, upper GI tissue for IL‐17 and human breast cancer tissue for E‐cadherin) and negative controls (non‐immune immunoglobulin G). Slides were coverslipped with a Leica CV5000 Coverslipper (Leica Microsystems, Inc., Buffalo Grove, IL) and imaged using a PanoramicScan 150 whole‐slide image scanner (3DHISTECH, Budapest, Hungary). Slides were counterstained with haematoxylin.

**Figure 1:**
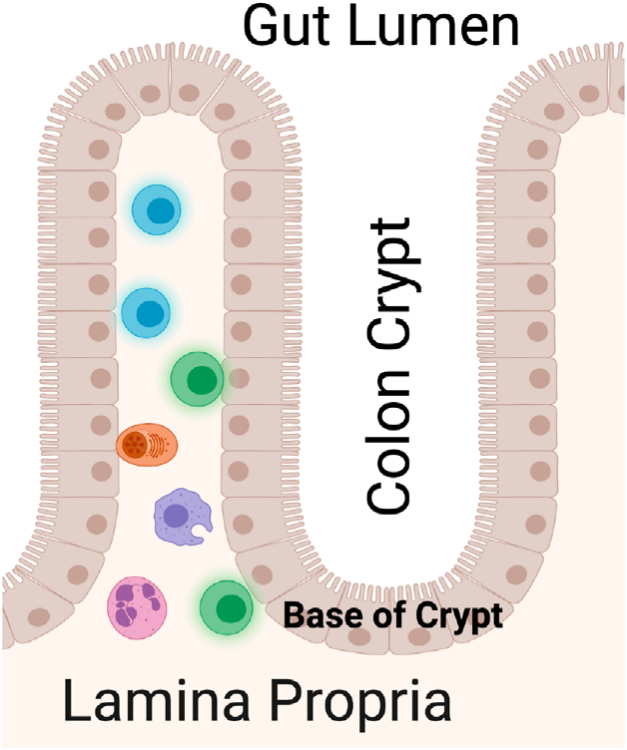
Key structural components of the rectal mucosa. Crypts are glands found in the epithelial lining of the small and large intestine. The basal portion of the crypt contains multipotent stem cells that form new epithelium. The lamina propria underlies the epithelium. The lumen refers to the gut lumen, which is the centre of the intestine. Adapted from “Rectum epithelium (enterocytes only),” by BioRender.com (2021). Retrieved from https://app.biorender.com/biorender‐templates.

As illustrated in Figure 1, key structural components of the rectal mucosa include the crypt epithelium, the crypt epithelial bases, the lamina propria, which houses a rich array of innate and adaptive immune cells, and the lumen, which refers to the centre of the intestine. To quantify biomarker labelling (“expression”) within the colon crypts or adjacent lamina propria, a precise image analysis scoring method was used as previously described [22]. A minimum of three hemicrypts or inter‐crypt lamina propria regions were scored per patient per visit per biomarker. Samples of blinded, previously scored slides were rescored to assess scorer reliability. Using these procedures, we analysed on average approximately 23 hemicrypts/biomarker/participant yielding a total of approximately 2396 hemicrypts analysed per biomarker across all participants. The intra‐class correlation coefficients for all biomarkers were ≥0.92.

### 2.3 Dual‐staining immunohistochemistry experiments

To investigate whether the source of IL‐17 and FOXP3 staining was CD4+ cells, dual‐staining IHC was conducted on a few specimens using a biotin‐free polymer system. Full methods are reported in the Supporting Information.

### 2.4 Microbiome sequencing

Rectal secretions were collected with a dacron swab during rigid sigmoidoscopy, and the microbiota was sequenced using 16S rRNA (V1–V2 region) methods as described in our prior study [15]. An operational taxonomic unit (OTU) table generated for the prior study was utilized to test associations with biomarker expression in this study as detailed below. Raw 16S rRNA sequences were placed in the NCBI Sequence Read Archive (SRA) under BioProject PRJNA322688.

### 2.5 Statistical analyses

**Figure 2:**
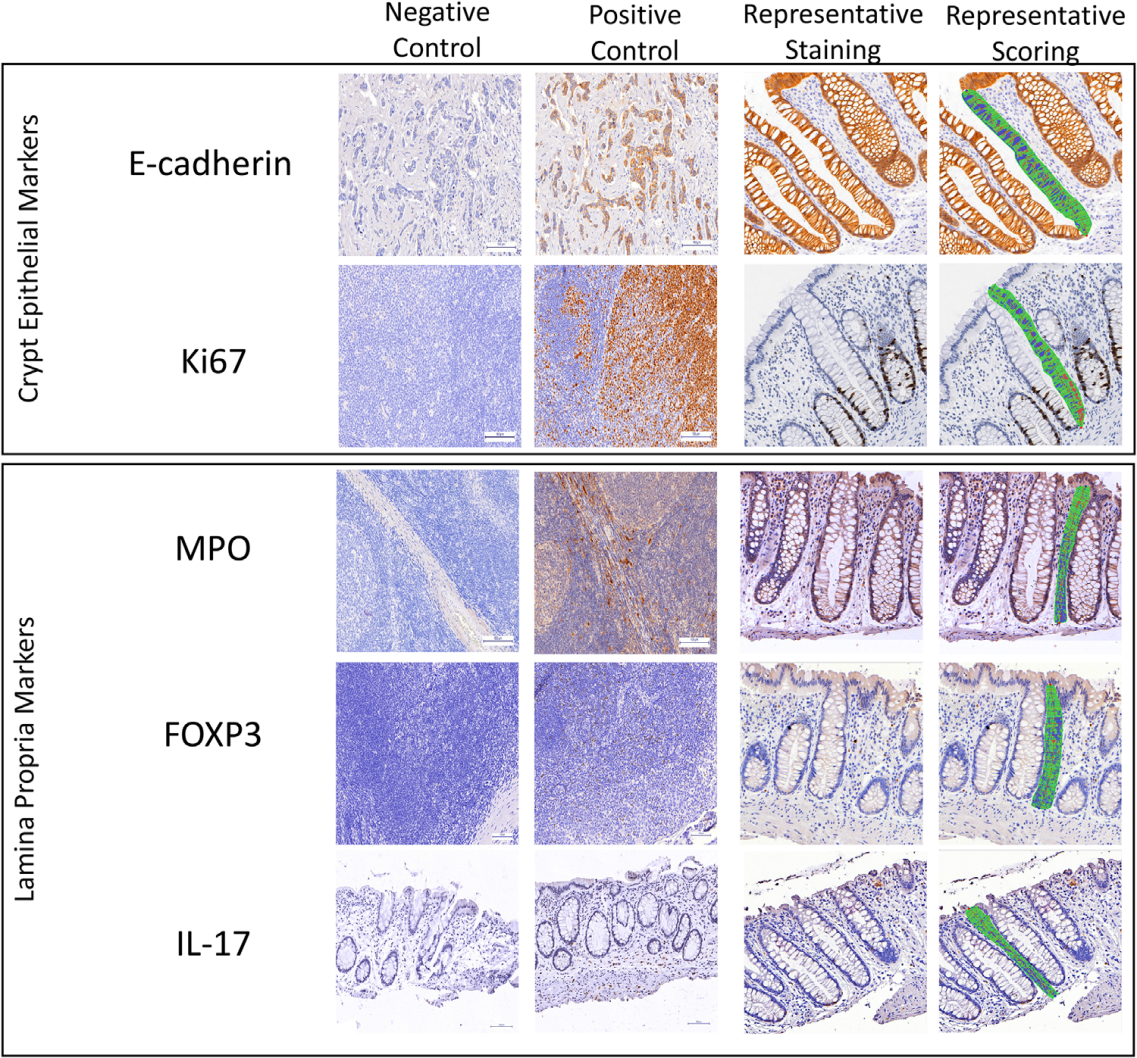
Representative immunohistochemical staining for rectal crypt epithelium markers E‐cadherin and Ki67 and stromal markers MPO, FOXP3 and IL‐17 within human colorectal mucosa. Tissue is from MSM engaging in CRAI. Positive control tissues are breast cancer tissue for E‐cadherin, tonsillar tissue for Ki67, MPO and FOXP3 and upper GI tissue for IL‐17 as described in the Methods section. Negative control staining was done with non‐immune IgG as described in the Methods section. All histologic sections were counterstained with haematoxylin. Images also depict aspects of image analyses to measure the optical densities of the biomarkers in colonic hemicrypts (epithelial markers) and in the areas adjacent to the hemicrypts (lamina propria markers) outlined in green and separated into 50 equidistant segments from the base of the crypt area to the lumen. A minimum of three regions were scored per biomarker, per participant visit.

Study group demographic and clinical characteristics were compared using the Wilcoxon rank‐sum test for continuous variables and Fisher's exact test for categorical variables. The primary outcome for the five biomarkers was the mean optical density along the scored hemicrypt length or adjacent lamina propria as outlined in Figure 2. The mean optical density was transformed by the natural logarithm to normalize the data for further analyses. Univariate correlations of biomarker expression with age, race, enema use and reported number of RAI partners in the last month were first examined using Spearman correlation coefficients. Because almost all MSM engaging in CRAI reported lubricant use, its use could not be examined. To assess longitudinal outcomes between MSM engaging in CRAI and controls, repeated measures analyses for each of the five biomarkers as the outcome were performed with a linear mixed model in R (Boston, MA) providing separate estimates of the means by study visit and study group after adjusting for age and race. Specifically, the initial model included five predictors: study group, visit number, interaction between study group and visit number, age and race. Since neither the interaction nor study visit terms were initially statistically significant, they were excluded in our final linear mixed model that produced an overall mean biomarker estimate for each study group by pooling observations from visits 1 and 2 as repeated measurements.

To evaluate associations of the microbiome with the five biomarkers measured using IHC and image analysis, we first fit a linear decomposition model (LDM) [23], adjusting for study group effect. LDM provides a single analysis path that includes global tests of any effect of the microbiome and tests of the effects of the relative abundance of individual OTUs while accounting for multiple testing by controlling the false discovery rate. As a comparison and confirmation, we applied PERMANOVA based on Bray–Curtis distance, which also provides a global test of significance, after dichotomizing biomarker expression levels at the median (i.e. high vs. low expression) [24]. Since *Prevotella* and *Bacteroides* were specific genera of interest in this study, we additionally fit linear regression models adjusted for study group to examine the association of the untransformed relative abundance of Prevotella or Bacteroides taxa (both family and genus‐level OTU) with each of the five biomarkers for hypothesis generation purposes.

### 2.6 Role of funding source

This study was funded by the National Institutes of Health. The funder had no role in the study design, collection, analysis and interpretation of data, report writing or decision to submit for publication.

## 3 RESULTS

Cohort demographics are presented in Table 1. Figure 2 displays representative images of rectal mucosal histologic sections immunohistochemically processed for the five biomarkers. The initial linear mixed model that included covariates for study visit and the study group*visit interaction demonstrated no substantial or statistically significant biomarker expression differences between visit 1 and 2 within either study group (Table 2), so they were excluded from the final model. The final linear mixed model examined overall differences between study groups controlling for race and age.

**Table 2:** Biomarker expression quantification using immunohistochemistry analysis within colonic crypts in MSM engaging in CRAI and men who never engaged in AI (controls)

	Initial model [1]						Final model [2]	
Biomarkers and study groups	*n*	Visit 1^a^, mean biomarker expression^b^ (95% CI)	*n*	Visit 2, mean biomarker expression (95% CI)	V2 versus V1 mean biomarker expression difference (V2–V1)	*p*‐Value	Group overall mean difference (95% CI)	*p*‐Value
FOXP3								
CRAI	32	4.24 (3.96, 4.52)	33	4.30 (4.06, 4.54)	0.06	0.57	0.04 (–0.29, 0.36)	0.83
Control	18	4.17 (3.92, 4.42)	14	4.33 (3.95, 4.70)	0.16	0.32		
MPO								
CRAI	38	6.14 (5.82, 6.47)	38	6.27 (5.95, 6.58)	0.13	0.29	**0.36 (–0.018, 0.69)**	**0.04**
Control	20	5.8 (5.52, 6.07)	16	5.92 (5.58, 6.25)	0.12	0.28		
Ki67								
CRAI	39	6.04 (5.73, 6.35)	38	6.19 (5.87, 6.52)	0.16	0.22	**0.46 (0.031, 0.88)**	**0.04**
Control	19	5.72 (5.35, 6.09)	16	5.58 (5.21, 5.94)	–0.14	0.49		
IL‐17								
CRAI	33	5.45 (4.90, 6.00)	33	5.51 (4.91, 6.10)	0.06	0.56	0.001 (–0.68, 0.69)	0.99
Control	18	5.49 (4.93, 6.05)	14	5.46 (4.88, 6.04)	–0.03	0.84		
E‐cadherin								
CRAI	32	8.61 (8.47, 8.75)	32	8.51 (8.34, 8.69)	–0.11	0.12	0.09 (–0.067, 0.24)	0.26
Control	16	8.58 (8.45, 8.72)	13	8.56 (8.40, 8.72)	–0.03	0.66		

*Note*: Bolded items are significant *p‐*values (<0.05).

Abbreviations: CI, confidence interval; CRAI, condomless receptive anal intercourse; E‐cadherin, epithelial cadherin; FOXP3, forkhead box P3; IL‐17, interleukin 17; Ki67, antigen Ki67; MPO, myeloperoxidase; V1, visit 1; V2, visit 2.

^a^ MSM did not engage in CRAI for >72 hours prior to visit 1 and did engage in CRAI <24 hours prior to visit 2.

^b^ Mean biomarker expression was measured as optical density and log‐transformed for analyses.

**Figure 3:**
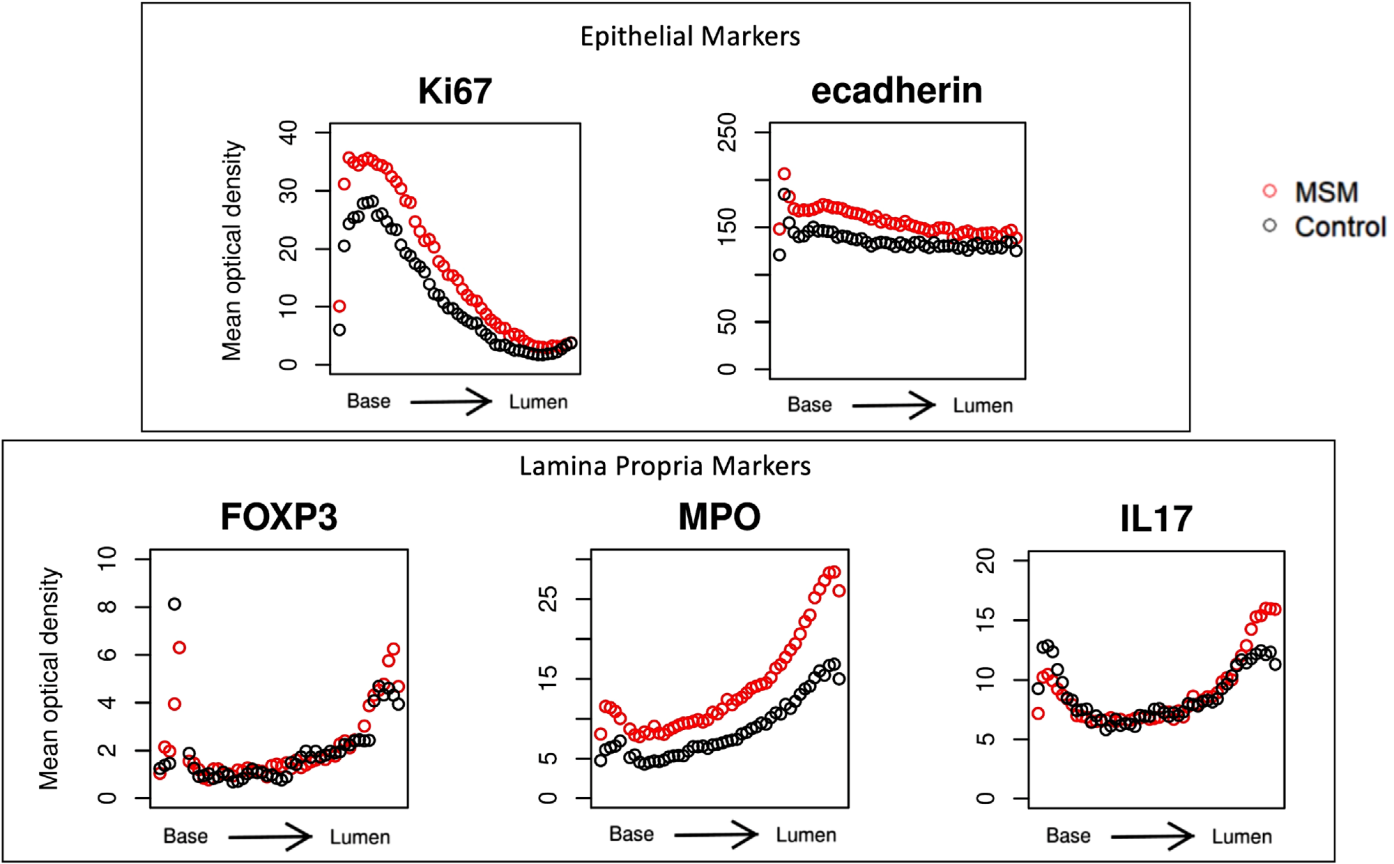
Distribution of lamina propria markers FOXP3, MPO and IL‐17, and epithelial markers Ki67 and E‐cadherin in the crypts of normal‐appearing rectal mucosa in MSM. Values for graphs were generated using automated immunohistochemistry and quantitative image analysis. Full‐length hemicrypts (crypt epithelium markers) and regions adjacent to full‐length hemicrypts (lamina propria markers) were analysed separately via tracing the region, followed by automated sectioning and quantification of the optical density of the immunohistochemically labelled biomarker within each section. Each hemicrypt was divided into 50 sub‐sections, denoted along the x‐axis, of average colonocyte width within which biomarker expression was quantified. Optical density values (y‐axis) were averaged across study visits for visual representation. MSM engaging in CRAI are represented by red circles and controls are represented by black circles.

The rectal mucosal epithelium of MSM engaging in CRAI had statistically significantly higher levels of proliferation relative to controls. Full‐length rectal hemicrypts of MSM engaging in CRAI demonstrated significantly higher Ki67 expression relative to controls (Table 2 and Figure 3; *p* = 0.04). Ki67 expression (calculated from estimates prior to log transformation) was overall 60% higher among MSM engaging in CRAI compared to controls. Ki67 and E‐cadherin expression distribution decreased from the base of the rectal crypts towards the colon lumen (Figure 3). There was no significant difference between E‐cadherin expression between study groups or significant univariate correlations between any of the epithelial biomarkers and age, race, enema use or the number of RAI partners in the last month (data not shown).

### 3.1 The rectal mucosal lamina propria of MSM engaging in CRAI was enriched for neutrophils relative to controls

We next examined MPO (neutrophils), IL‐17 (pro‐inflammatory mucosal cells) and FOXP3 (regulatory T cells) expression in the lamina propria tissue adjacent to the rectal crypts (Figure 2). In the final linear mixed model controlling age and race, MPO expression in the lamina propria was significantly higher among MSM engaging in CRAI than among controls (Table 2 and Figure 3; *p* = 0.04). Overall, MPO expression (calculated from estimates prior to log transformation) was 41% higher among MSM engaging in CRAI than among controls. Expression of all three cellular biomarkers increased from the crypt base towards the gut lumen, likely reflecting higher environmental and microbial exposure at the mucosal surface–lumen interface (Figure 3). There were no significant IL‐17 or FOXP3 expression differences between the two study groups and no significant univariate correlations between the expression of any of the cellular biomarkers and age, race, enema use or the number of RAI partners in the last month (data not shown).

**Figure 4:**
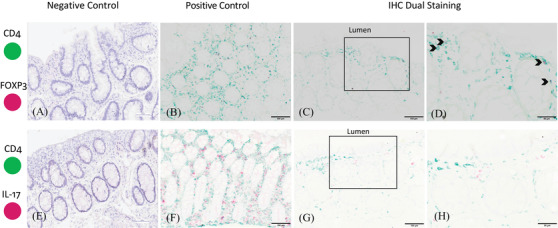
Representative images of dual immunohistochemical detection of the biomarkers in rectal mucosal tissue. Tissue is from MSM engaging in CRAI. The biomarkers were CD4 (green) and FOXP3 (red; panels A–D) and CD4 (green) and IL‐17 (red; panels E–H). Black arrows (D) show the colocalization of CD4 and FOXP3 expression. Panels D and H are enlargements of the outlined boxes in panels C and G, respectively. Scale bars, 100 μm (A–C and E–G) or 50 μm (D and H).

Our dual‐staining IHC experiments demonstrated that FOXP3 co‐localized with CD4 but IL‐17 did not (Figure 4), suggesting that the FOXP3‐positive cells represent T_reg_ cells. However, the source of cellular IL‐17 does not appear to be Th17 cells but may have been γδ T cells, NK cells and/or NK‐T cells.

**Figure 5:**
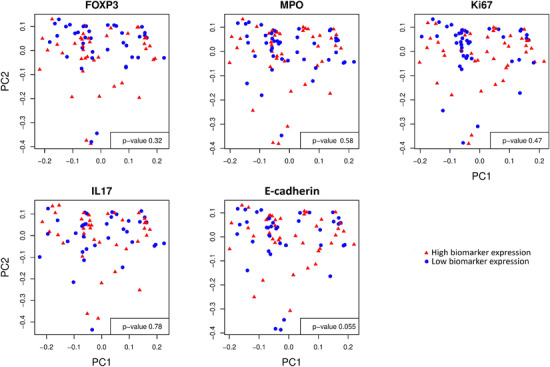
Principal coordination analysis (PCoA) plots for Bray–Curtis distance, a measure of beta diversity of the microbiome, showing no separation of the low (blue circles) and high (red triangles) biomarker expression groups, which were dichotomized at median expression values. *p*‐Values represent PERMANOVA test of significance. PC1, or principal coordinate 1, refers to the direction that captures the most variation in microbiome beta diversity. PC2, or principal coordinate 2, refers to the direction that captures the second‐most variation in microbiome beta diversity.

The rectal mucosa microbiota was not clearly associated with biomarker expression, but further investigations are warranted, particularly for microbiota associations with E‐cadherin. Global tests of significance for associations between the microbiome and each of the five evaluated mucosal biomarkers approached significance only for E‐cadherin expression (LDM *p* = 0.06, PERMANOVA *p* = 0.06; Figure 5). As this is a global test of significance inclusive of the entire microbiome, there is no directionality associated with the effect. No individual genera were statistically significantly associated with E‐cadherin expression after adjustment for multiple comparisons in the LDM models. We also did not find consistent associations of the *Prevotellaceae* or *Bacteroidaceae* taxa with the five biomarkers in our hypothesis‐generating linear regression model analyses.

## 4 DISCUSSION

These data support and add mechanistic detail to our prior discovery of a distinct rectal mucosal immune environment among MSM engaging in CRAI compared to men who do not engage in AI [15]. Using a new method, automated IHC with quantitative image analysis, we were able to examine the distribution of inflammation‐related immunologic biomarkers in the rectal mucosa, a site particularly susceptible to HIV transmission. We were able to conduct a spatial examination of five biomarkers in the rectal epithelium and lamina propria from crypt base to gut lumen, where HIV and other pathogens first encounter the mucosal surface.

We found that MSM engaging in CRAI demonstrated significantly higher epithelial cell proliferation and neutrophil abundance compared to controls independent of CRAI timing or other examined demographic or risk behaviours. We also found that pro‐inflammatory neutrophils, IL‐17‐expressing cells and anti‐inflammatory T_regs_ were enriched towards the mucosal luminal surface, while proliferating epithelial cells and the adherens junction protein E‐cadherin were enriched in the epithelial crypt base. These data further underscore our hypothesis that any combination of subclinical microtrauma during CRAI, semen exposure or product use during intercourse (e.g. lubricants and enemas) may be associated with chronic rectal immunologic changes that could facilitate HIV or sexually transmitted infection acquisition or affect response to biomedical prevention interventions, including candidate HIV vaccines.

The increased rectal mucosal neutrophil abundance in MSM engaging in CRAI demonstrated in this study and upregulated rectal expression of genes involved in neutrophil function in MSM engaging in CRAI demonstrated in our 2017 study together present convincing evidence of a subclinical inflammatory mucosal response to CRAI. Neutrophils have a complex role in intestinal homeostasis. They are the first immune cells to respond to injured tissue [25] and are responsible for clearing harmful pathogens. Neutrophils can release elastase, which disrupts E‐cadherin‐based cell–cell junctions, resulting in the loss of epithelial barrier function [26]. Importantly, these changes may not lead to lower E‐cadherin expression overall but rather uneven marker distribution. Furthermore, barrier integrity disruption can lead to secondary inflammation by allowing bacterial movement from the lumen and by homing large numbers of Th cells, which are potential HIV targets, into inflamed regions [16, 27]. In this context, our discovery of increased rectal mucosal neutrophil abundance and activity in MSM engaging in CRAI could increase HIV transmission risk in two ways: both by compromising intestinal epithelial barrier function as a result of heightened mucosal inflammation and by increasing HIV target cell recruitment to the area of highest transmission risk.

We did not note a significant difference in E‐cadherin expression between study groups but may have been underpowered to detect the small difference near the crypt base seen in our biomarker distribution graphs. It is important to note, however, that staining for E‐cadherin alone does not provide a complete understanding of barrier integrity. E‐cadherin belongs to a large family of adherens junction proteins that, together with tight junction proteins, play critical roles in maintaining the colonic crypt architecture and functions [28]. Junction efficacy is crucial for normal intestinal physiology, and perturbations in these connections can result in adverse inflammation or gut dysbiosis [29]. Our results call for further research into rectal mucosal epithelial barrier integrity and expression and distribution of key adherens and tight junction proteins among MSM engaging in CRAI.

The intestinal mucosal epithelial layer is also dynamic. Epithelial cells are continually renewed by pluripotent Lgr5+ crypt base columnar (CBC) stem cells that reside in intestinal crypt bases [30]. In our study, MSM engaging in CRAI had significantly higher crypt epithelial cell proliferation (demonstrated by higher Ki67 expression) than controls. There is great flexibility in the regenerative capacity of the intestinal epithelium, and different extrinsic or intrinsic factors can modify this process of homeostatic epithelial self‐renewal. The physiological response to intestinal mucosal injury is characterized by an initial phase of inflammation followed by epithelial migration, restructuring and epithelial cell proliferation [30]. Through cell division, additional cells are added to the mucosal milieu to aid in repair. Other studies point to the importance of the inflammatory cell milieu in regulating CBC proliferation. In our previous study, genes implicated in mucosal injury were upregulated in MSM engaging in CRAI [15]. Therefore, it is likely that the increased epithelial cell proliferation seen in the current study is the normal, physiologic response to subclinical CRAI‐associated mucosal inflammation. It is unclear whether increased epithelial cell proliferation is associated with adverse health outcomes [31, 32] or mucosal pathogen transmission.

Our original intention in staining for IL‐17 and FOXP3 was to evaluate the contributions of Th17 and T_reg_ cells in the lamina propria given our prior flow‐cytometry‐based finding that MSM engaging in CRAI demonstrated more rectal mucosal Th17 cells than did controls. Th17 cells are known targets for HIV infection and promote recruitment of pro‐inflammatory immune cells, including neutrophils, to mucosal tissues during early infection, which further supports viral replication [27, 33]. In addition, the ratio of Th17/T_reg_ cells in gut mucosal tissues may be an important driver of mucosal inflammation [34, 35]. T_reg_ cells modulate the immune response, suppress mucosal tissue inflammation and may facilitate healing [12, 36]. We did not detect a difference between study groups in IL‐17 or T_reg_ expression, but we did find increasing concentrations of these cells towards the rectal lumen where pathogen exposure, including HIV, is expected to first occur. A multitude of innate and adaptive immune cells can also produce IL‐17, notably CD8+ T cells, MAIT cells, NK cells, NK‐T cells, γδ 17 T cells and innate lymphoid cells [37, 38]. These are the likely source of cellular IL‐17 seen in our study as the dual staining experiments did not show co‐localization of CD4 and IL‐17 staining. Given the importance of this cytokine in mucosal defence maintenance and epithelial barrier function preservation, it is likely that IL‐17‐producing cells contributed to our findings of higher rectal epithelial cell proliferation and neutrophil abundance in MSM engaging in CRAI despite not finding a difference between study groups.

A limitation of our study is the inability to link the immunologic data with clinical outcomes, including HIV seroconversion. This would require large, longitudinal cohorts or clinical trials among MSM that incorporate mucosal sampling and HIV incidence outcomes, which may not be feasible. We are currently exploring other approaches, including utilizing novel rectal mucosal HIV explant challenges as a proxy for HIV transmission and replication [39]. Because we selected our study biomarkers based on hypotheses generated from our prior work (e.g. MPO and IL‐17) and existing expertise in our automated IHC/quantitative image analysis laboratory (e.g. Ki67 and E‐cadherin), we could not fully characterize the rectal mucosal epithelial cell junctions or all cellular populations of interest. This will require further investigation. Finally, our study's modest sample size limited the statistical power to detect small differences.

## 5 CONCLUSIONS

Our findings make important contributions to a comprehensive understanding of the ways in which CRAI affects the rectal mucosal immune environment, which is essential to the development of effective biomedical HIV prevention interventions. Increased neutrophil abundance and epithelial cell proliferation in the rectal mucosa of MSM engaging in CRAI, in addition to our previous findings of immunologic differences, including increased Th17 cells, proliferating CD8 cells, pro‐inflammatory cytokine production from CD8 cells, gene signatures associated with mucosal inflammation and subclinical injury/repair, and a microbiota enriched for *Prevotella* over *Bacteroides*, suggest a plausible effect on subsequent HIV transmission risk and response to candidate HIV vaccines. Also, our data suggest that prevention interventions that reduce rectal mucosal inflammation or capitalize on the presence of a specific inflammatory mechanism (e.g. neutrophil response) at the time of mucosal HIV exposure deserve further exploration. Future research must carefully consider the human, sexual contexts that influence the mucosal immune environment and their potential impact on HIV outcomes.

## COMPETING INTERESTS

All authors report no competing interests with this work.

## AUTHOR'S CONTRIBUTIONS

CFK conceived the project, supervised all human subjects and laboratory research, verified all data and findings and wrote the manuscript. IP wrote the manuscript. RY performed laboratory assays, data analyses and provided critical review of the manuscript. ZZ performed data analyses, wrote and provided critical review of the manuscript. VEVD wrote and provided critical review of the manuscript. SG performed dual IHC‐staining experiments and provided critical review of the manuscript. RRA provided guidance on immunologic assays and interpretation and critical review of the manuscript. VF assisted with IHC data analyses and interpretation and provided critical review of the manuscript. CSK performed microbiome sequencing assays and provided critical review of the manuscript. TJBD analysed microbiome data and provided critical review of the manuscript. YH performed data analyses and provided critical review of the manuscript. CGA assisted with data analyses and provided critical review of the manuscript. PSS contributed to the construct and conduct of the human subjects cohort and provided critical review of the manuscript. RMB oversaw the development and application of the automated immunohistochemistry with quantitative image analysis protocols, assisted with interpretation of data and analyses, and provided critical review of the manuscript.

## FUNDING

The study received grants K23 AI108335 (CFK), U19 AI109633 (RRA.), The Emory Center for AIDS Research P30AI050409 and The Atlanta Clinical and Translational Science Institute UL1TR000454.

## Supporting information

Supporting Information
**Additional file S1**: Methods describing dual staining experiments.

## Data Availability

Study protocol, informed consent and de‐indentified data collected for this study, including individual, participant‐level data and data dictionary, can be made available upon request after publication with a signed data‐use agreement. Please send email request to colleen.kelley@emory.edu.

## References

[1] Sullivan PS , Salazar L , Buchbinder S , Sanchez TH . Estimating the proportion of HIV transmissions from main sex partners among men who have sex with men in five US cities. AIDS. 2009;23:1153–62. 19417579 10.1097/QAD.0b013e32832baa34

[2] Centers for Disease Control and Prevention . HIV surveillance report. 2018.

[3] Baggaley RF , White RG , Boily M‐C . HIV transmission risk through anal intercourse: systematic review, meta‐analysis and implications for HIV prevention. Int J Epidemiol. 2010;39:1048–63. 20406794 10.1093/ije/dyq057 PMC2929353

[4] Hladik F , Mcelrath MJ . Setting the stage: host invasion by HIV. Nat Rev Immunol. 2008;8:447–57. 18469831 10.1038/nri2302 PMC2587276

[5] Mcelrath MJ , Smythe K , Randolph‐Habecker J , Melton KR , Goodpaster TA , Hughes SM , et al. Comprehensive assessment of HIV target cells in the distal human gut suggests increasing HIV susceptibility toward the anus. J Acquir Immune Defic Syndr. 2013;63:263–71. 23392465 10.1097/QAI.0b013e3182898392 PMC3683090

[6] Poles MA , Elliott J , Taing P , Anton PA , Chen ISY . A preponderance of CCR5(+) CXCR4(+) mononuclear cells enhances gastrointestinal mucosal susceptibility to human immunodeficiency virus type 1 infection. J Virol. 2001;75:8390–9. 11507184 10.1128/JVI.75.18.8390-8399.2001 PMC115084

[7] Levinson P , Kaul R , Kimani J , Ngugi E , Moses S , Macdonald KS , et al. Levels of innate immune factors in genital fluids: association of alpha defensins and LL‐37 with genital infections and increased HIV acquisition. AIDS. 2009;23:309–17. 19114868 10.1097/QAD.0b013e328321809c

[8] Hirbod T , Kong X , Kigozi G , Ndyanabo A , Serwadda D , Prodger JL , et al. HIV acquisition is associated with increased antimicrobial peptides and reduced HIV neutralizing IgA in the foreskin prepuce of uncircumcised men. PLoS Pathog. 2014;10:e1004416. 25275513 10.1371/journal.ppat.1004416 PMC4183701

[9] Prodger JL , Gray RH , Shannon B , Shahabi K , Kong X , Grabowski K , et al. Chemokine levels in the penile coronal sulcus correlate with HIV‐1 acquisition and are reduced by male circumcision in Rakai, Uganda. PLoS Pathog. 2016;12:e1006025. 27898732 10.1371/journal.ppat.1006025 PMC5127584

[10] Arnold KB , Burgener A , Birse K , Romas L , Dunphy LJ , Shahabi K , et al. Increased levels of inflammatory cytokines in the female reproductive tract are associated with altered expression of proteases, mucosal barrier proteins, and an influx of HIV‐susceptible target cells. Mucosal Immunol. 2016;9:194–205. 26104913 10.1038/mi.2015.51

[11] Brockmann L , Giannou A , Gagliani N , Huber S . Regulation of TH17 cells and associated cytokines in wound healing, tissue regeneration, and carcinogenesis. Int J Mol Sci. 2017;18:1033. 10.3390/ijms18051033 PMC5454945 28492497

[12] Nosbaum A , Prevel N , Truong H‐A , Mehta P , Ettinger M , Scharschmidt TC , et al. Cutting edge: regulatory T cells facilitate cutaneous wound healing. J Immunol. 2016;196:2010–4. 26826250 10.4049/jimmunol.1502139 PMC4761457

[13] Maric D , Grimm WA , Greco N , McRaven MD , Fought AJ , Veazey RS , et al. Th17 T cells and immature dendritic cells are the preferential initial targets after rectal challenge with an SIV‐based replication‐defective dual‐reporter vector. J Virol. 2021;95(19):e0070721. 34287053 10.1128/JVI.00707-21 PMC8428389

[14] Capaldo CT , Nusrat A . Claudin switching: physiological plasticity of the tight junction. Semin Cell Dev Biol. 2015;42:22–9. 25957515 10.1016/j.semcdb.2015.04.003

[15] Kelley CF , Kraft CS , De Man T , Duphare C , Lee H‐W , Yang J , et al. The rectal mucosa and condomless receptive anal intercourse in HIV‐negative MSM: implications for HIV transmission and prevention. Mucosal Immunol. 2017;10:996–1007. 27848950 10.1038/mi.2016.97 PMC5433931

[16] Kolaczkowska E , Kubes P . Neutrophil recruitment and function in health and inflammation. Nat Rev Immunol. 2013;13:159–75. 23435331 10.1038/nri3399

[17] Pérez MM , Martins LMS , Dias MS , Pereira CA , Leite JA , Gonçalves ECS , et al. Interleukin‐17/interleukin‐17 receptor axis elicits intestinal neutrophil migration, restrains gut dysbiosis and lipopolysaccharide translocation in high‐fat diet‐induced metabolic syndrome model. Immunology. 2019;156:339–55. 30472727 10.1111/imm.13028 PMC6418416

[18] Haaland RE , Fountain J , Hu Y , Holder A , Dinh C , Hall L , et al. Repeated rectal application of a hyperosmolar lubricant is associated with microbiota shifts but does not affect PrEP drug concentrations: results from a randomized trial in men who have sex with men. J Int AIDS Soc. 2018;21:e25199. 30378274 10.1002/jia2.25199 PMC6207839

[19] Dezzutti CS , Brown ER , Moncla B , Russo J , Cost M , Wang L , et al. Is wetter better? An evaluation of over‐the‐counter personal lubricants for safety and anti‐HIV‐1 activity. PLoS One. 2012;7:e48328. 23144863 10.1371/journal.pone.0048328 PMC3492332

[20] Javanbakht M , Stahlman S , Pickett J , Leblanc M‐A , Gorbach PM . Prevalence and types of rectal douches used for anal intercourse: results from an international survey. BMC Infect Dis. 2014;14:95. 24555695 10.1186/1471-2334-14-95 PMC4015843

[21] Rolle C‐PM , Bolton MD , Kelley CF . Use of a prospective sex diary to study anal lubricant and enema use among high risk men who have sex with men—implications for human immunodeficiency virus prevention. Sex Transm Dis. 2016;43:476–8. 27419813 10.1097/OLQ.0000000000000473 PMC4948947

[22] Liu S , Barry EL , Baron JA , Rutherford RE , Seabrook ME , Bostick RM . Effects of supplemental calcium and vitamin D on the APC/beta‐catenin pathway in the normal colorectal mucosa of colorectal adenoma patients. Mol Carcinog. 2017;56:412–24. 27254743 10.1002/mc.22504 PMC5586148

[23] Hu Y‐J , Satten GA . Testing hypotheses about the microbiome using the linear decomposition model (LDM). Bioinformatics. 2020;36:4106–15. 32315393 10.1093/bioinformatics/btaa260 PMC8453243

[24] Mcardle BH , Anderson MJ . Fitting multivariate models to community data: a comment on distance‐based redundancy analysis. Ecology. 2001;82:290–7.

[25] Engelhardt E , Toksoy A , Goebeler M , Debus S , Bröcker E‐B , Gillitzer R . Chemokines IL‐8, GROalpha, MCP‐1, IP‐10, and Mig are sequentially and differentially expressed during phase‐specific infiltration of leukocyte subsets in human wound healing. Am J Pathol. 1998;153:1849–60. 9846975 10.1016/s0002-9440(10)65699-4 PMC1866330

[26] Ginzberg HH , Cherapanov V , Dong Q , Cantin A , Mcculloch CAG , Shannon PT , et al. Neutrophil‐mediated epithelial injury during transmigration: role of elastase. Am J Physiol Gastrointest Liver Physiol. 2001;281:G705–17. 11518683 10.1152/ajpgi.2001.281.3.G705

[27] Stieh DJ , Matias E , Xu H , Fought AJ , Blanchard JL , Marx PA , et al. Th17 cells are preferentially infected very early after vaginal transmission of SIV in macaques. Cell Host Microbe. 2016;19:529–40. 27078070 10.1016/j.chom.2016.03.005 PMC4841252

[28] Valizadeh A , Karayiannakis AJ , el‐Hariry I , Kmiot W , Pignatelli M . Expression of E‐cadherin‐associated molecules (alpha‐, beta‐, and gamma‐catenins and p120) in colorectal polyps. Am J Pathol. 1997;150:1977–84. 9176391 PMC1858309

[29] Pearce SC , Al‐Jawadi A , Kishida K , Yu S , Hu M , Fritzky LF , et al. Marked differences in tight junction composition and macromolecular permeability among different intestinal cell types. BMC Biol. 2018;16:19. 29391007 10.1186/s12915-018-0481-z PMC5793346

[30] Leoni G , Neumann P‐A , Sumagin R , Denning TL , Nusrat A . Wound repair: role of immune–epithelial interactions. Mucosal Immunol. 2015;8:959–68. 26174765 10.1038/mi.2015.63 PMC4916915

[31] Choi HJ , Do KH , Park J‐H , Kim J , Yu M , Park S‐H , et al. Early epithelial restitution by nonsteroidal anti‐inflammatory drug‐activated gene 1 counteracts intestinal ulcerative injuries. J Immunol. 2016;197:1415–24. 27421482 10.4049/jimmunol.1501784

[32] Dubeykovskaya Z , Dubeykovskiy A , Solal‐Cohen J , Wang TC . Secreted trefoil factor 2 activates the CXCR4 receptor in epithelial and lymphocytic cancer cell lines. J Biol Chem. 2009;284:3650–62. 19064997 10.1074/jbc.M804935200 PMC2635042

[33] Hensley‐Mcbain T , Klatt NR . The dual role of neutrophils in HIV infection. Curr HIV/AIDS Rep. 2018;15:1–10. 29516266 10.1007/s11904-018-0370-7 PMC6086572

[34] Falivene J , Ghiglione Y , Laufer N , Socías ME , Holgado MP , Ruiz MJ , et al. Th17 and Th17/Treg ratio at early HIV infection associate with protective HIV‐specific CD8(+) T‐cell responses and disease progression. Sci Rep. 2015;5:11511. 26099972 10.1038/srep11511 PMC4477236

[35] Caruso MP , Falivene J , Holgado MP , Zurita DH , Laufer N , Castro C , et al. Impact of HIV‐ART on the restoration of Th17 and Treg cells in blood and female genital mucosa. Sci Rep. 2019;9:1978. 30760809 10.1038/s41598-019-38547-1 PMC6374372

[36] Arpaia N , Green JA , Moltedo B , Arvey A , Hemmers S , Yuan S , et al. A distinct function of regulatory T cells in tissue protection. Cell. 2015;162:1078–89. 26317471 10.1016/j.cell.2015.08.021 PMC4603556

[37] Schön MP . The plot thickens while the scope broadens: a holistic view on IL‐17 in psoriasis and other inflammatory disorders. Exp Dermatol. 2014;23:804–6. 25219805 10.1111/exd.12541

[38] Lin AM , Rubin CJ , Khandpur R , Wang JY , Riblett M , Yalavarthi S , et al. Mast cells and neutrophils release IL‐17 through extracellular trap formation in psoriasis. J Immunol. 2011;187:490–500. 21606249 10.4049/jimmunol.1100123 PMC3119764

[39] Smith SA , Murray PM , Amancha PK , Grimsley‐Ackerley C , Amara RR , Kelley CF . Opposing associations of NK and MZB cells in rectal explant model of HIV infection. Conference on Retroviruses and Opportunistic Infections. 2020.

