# Supplementary material for: Condomless receptive anal intercourse is associated with markers of mucosal inflammation in a cohort of men who have sex with men in Atlanta, Georgia
Source: J Int AIDS Soc. 2021 Dec 15;24(12):e25859. doi: 10.1002/jia2.25859 (PMC8673926; doi:10.1002/jia2.25859)
Supplement: Supplementary file 1 — Supporting Information Additional file S1: Methods describing dual staining experiments. [file JIA2-24-e25859-s001.docx]

**Supplementary methods**

**Dual staining immunohistochemistry experiments.** To investigate the cellular source of IL-17 and FOXP3 expression, dual staining IHC experiments were carried out on a few specimens. Double staining on histologic sections of intestine was performed using a biotin-free polymer system. The paraffin-embedded sections were subjected to deparaffinization in xylene, rehydration in a graded series of ethanol, and rinsed with double distilled water. Antigen retrieval was performed by immersing sections in Triology (Cell Marque**)** at 125˚C for 30 seconds in a steam pressure decloaking chamber (Biocare Medical) followed by blocking with Background Sniper Reagent (Biocare Medical) for 10 minutes. The sections were incubated with mouse anti-human/Primate IL-17 (clone # 41809; R & D Systems) and rabbit anti-CD4 (clone EPR6855; Abcam) overnight at 4°C, followed by a double detection polymer system (Mach 2 Double Stain 2, Biocare Medical). Labeled antibodies were visualized by development of the chromogen (Warp Red and/or Vina Green Chromogen Kits; Biocare Medical). Digital images of the histologic sections were randomly captured at 100 × and 200 × magnification with an Olympus BX43 microscope equipped with a digital camera (DP26, Olympus) and evaluated using Cellsens digital imaging software 1.11 (Olympus).
